# Global commitment to combat antimicrobial resistance

**DOI:** 10.1590/0102-311XEN190924

**Published:** 2025-04-11

**Authors:** Claudia Chamas

**Affiliations:** 1 Centro de Desenvolvimento Tecnológico em Saúde, Fundação Oswaldo Cruz, Rio de Janeiro, Brasil.; 2 Instituto Nacional de Ciência e Tecnologia de Inovação em Doenças de Populações Negligenciadas, Rio de Janeiro, Brasil.

## Introduction

Antimicrobial resistance occurs when microorganisms (e.g., bacteria, viruses, fungi, parasites) no longer respond to the medicines formulated to eliminate them, making these treatments ineffective at containing infections, and affecting medical procedures that rely on these therapies to reduce the risk of complications ^1^. The matter requires a more powerful or costly approach. In the most severe cases, infections can even become untreatable, leading to deaths or long-term disabilities. This is a threat to global health, as the phenomenon does not know borders. Furthermore, it hampers meeting the Sustainable Development Goals, especially those related to health, poverty reduction, food security, and the environment. Factors such as lack of access to drinking water, sanitation and hygiene, poor infection control in health facilities, difficulty in accessing diagnostics, medication, and vaccines, aggravate the situation, particularly in low- and middle-income countries.

Annually, about 7.7 million people die due to bacterial infections, and 4.95 million deaths are related to antibiotic-resistant pathogens. Low- and middle-income countries endure much of the burden of antimicrobial resistance ^2^. To address the situation, on September 26th, 2024, in New York (United States), the 79th session of the United Nations (UN) General Assembly held the second High-Level Meeting on Antimicrobial Resistance. The meeting represented a renewed effort and an important signal from countries in the face of the growing threats posed by antimicrobial resistance ^2^. On the occasion, global leaders endorsed the *Political Declaration of the High-Level Meeting on Antimicrobial Resistance*. The intergovernmental negotiations that preceded this Declaration were co-facilitated by Malta and Barbados ^3^.

The 2024 Declaration is not the first of its kind, many other international initiatives that have helped to reach the current level of alert and scope can be mentioned, namely: (i) in 2015, the *Global Action Plan on Antimicrobial Resistance*, which was adopted by the World Health Assembly, clarified the scenario and strategies that should be addressed via local and global actions ^4^; (ii) in 2016, a State leaders’ initiative at the UN − *Political Declaration of the High-Level Meeting of the General Assembly on Antimicrobial Resistance* ^5^ ^,^ ^6^ − signaled the urgency to contain the spread of antimicrobial-resistant pathogens; (iii) in 2017, the Interagency Coordination Group on Antimicrobial Resistance was set up − convened by the General-Secretary of the UN, which created recommendations to ensure sustained global action on antimicrobial resistance, including improved regulation, sustained One Health response and investments in innovation ^7^; and (iv) in 2017, the G20 Leaders’ Declaration - *Shaping an Interconnected World* - stressed the importance of promoting research and development, especially for priority pathogens identified by the World Health Organization (WHO) and tuberculosis ^8^.

Despite this relevant set of policies, plans, and declarations, humanity is far from having adequate solutions. Resistance compromises the effectiveness of antimicrobials that are essential for performing surgeries and managing diseases. Coordinated global action is one of the most effective ways to increase awareness and surveillance on this topic, which is a challenge to health, food security, and development. The 2024 Declaration establishes a significant commitment to leverage actions and monitoring, in addition to mobilizing investments, which are currently insufficient, especially for poorer countries.

The World Bank estimates that an annual USD 9 billion is needed for low- and middle-income countries. The intergovernmental institution South Centre proposes a global plan for mobilizing sustainable financing for antimicrobial resistance, aiming at the implementation of national action plans and budgeting for developing countries ^9^ ^,^ ^10^.

## Key points of the high-level meeting political declaration on antimicrobial resistance

The Declaration involves broad commitments on key aspects of fighting antimicrobial resistance. Global leaders have committed to reducing deaths associated with the problem by 10% by 2030, in addition to considering its “multifaceted and transversal nature” ^5^. Other relevant commitments include:

(i) Governance: by 2030, all countries should develop/update and implement multi-sectoral national action plans on antimicrobial resistance; calls on quadripartite organizations (WHO, Food and Agriculture Organization of the United Nations, World Organization for Animal Health, and UN Environment Program) to update the Global Action Plan on Antimicrobial Resistance by 2026; the Quadripartite Joint Secretariat on Antimicrobial Resistance should be formalized as the central coordination mechanism; the Secretariat’s cooperation with relevant multilateral organizations, such as the World Bank, the United Nations Children’s Fund, and the World Customs Organization, is encouraged; and quadripartite organizations are invited to establish an independent evidence panel for action against antimicrobial resistance by 2025.

(ii) Funding: agree on sustainable funding for implementation of national action plans; commit to sustainable funding for the implementation of national action plans on antimicrobial resistance; strengthen funding via existing institutions and mobilize resources via national, bilateral, and multilateral channels, especially for developing countries; and encourage existing funding mechanisms (e.g., World Bank, Global Fund to Fight AIDS, Tuberculosis and Malaria, Global Alliance for Vaccines and Immunization, etc.) to facilitate access to funding or expand its scope, including investments in effective antimicrobials, vaccines, research and development, diagnostics, among others.

(iii) Access: ensure equitable access to antimicrobials, vaccines, and diagnostics in developing countries, in line with global lists of essential medicines (e.g., WHO list of essential medicines).

(iv) Human health: ensure that national infection prevention and control programs are properly working via the implementation of the WHO Global Strategy on Infection Prevention and Control of 2023, the Immunization Agenda 2030, the Water, Sanitation, and Hygiene Strategy of the WHO 2018-2025, and the Global Action Plan on Patient Safety of the WHO 2021-2030, aiming to achieve targets such as 100% of countries with basic water, sanitation, hygiene, and waste services in all health facilities and 90% of countries meeting all the WHO minimum requirements for infection prevention and control programs at the national level by 2030; promote the timely and equitable provision of quality and affordable vaccines, diagnostics, and treatments, including antimicrobials, and ensure their appropriate use; and ensure, by 2030, that the use of antibiotics from the WHO access group is extended regarding the 2023 global target.

(v) Animal health and agriculture: efforts to reduce, by 2030, the number of antimicrobials used globally in the agri-food system compared to the current level, considering investments in animal and plant health to prevent and control infections, reducing the inappropriate use of antimicrobials.

(vi) Research and development, innovation, and manufacturing: promote funding for research and development to address antimicrobial resistance, as well as public-private sector partnerships, recognizing the need to increase public health research and development based on equity and accessibility; promote technology transfer and the know-how and encourage innovation and commitments to voluntary licensing, when possible, in agreements in which public funding has been invested in antimicrobial research, to strengthen local and regional manufacturing capacities and effective access to vaccines, therapies, diagnostics, and essential supplies; increase price transparency for medication, vaccines, medical devices, diagnostics, assistive products, cell- and gene-based therapies, and other health technologies across the value chain; support developing countries to strengthen local production of vaccines, medication, diagnostics, and other health technologies to facilitate equitable access, recognizing that high prices prevent the fight against antimicrobial resistance.

## Innovation and production: prospects for the near future

The last aspect is of special importance for emerging countries such as Brazil. The COVID-19 pandemic has shown us how having local innovation structures and local production are important not only for the densification of the pharmaceutical production chain, but to reduce vulnerabilities in the context of input and product restrictions and disruptions in global supply chains. In times of serious health crisis, robust and resilient systems save lives. Going back in time a bit, we remember what happened during the Falklands War in 1982. Argentina suffered a cut in the supply of strategic products such as antibiotics. Brazil could send shipments of products, because of the domestic manufacturing ^11^. There is no shortage of examples, even without wars or pandemics, to show us how much the production capacity of antibiotics and other products is essential for the country’s safety. Brazil needs to be prepared to face antimicrobial resistance and other adverse situations. In this context, State investment, global coordination, and local and international cooperation cannot be underestimated, aiming to increase the range of affordable options for treatments, vaccines, and diagnostics.

Although the set of interventions is broader and preventive actions, adequate protocols, and rational use of antibiotics, health care, surveillance, and improvement of water and sewage infrastructure can save countless lives and contribute to the mitigation of resistance, detection, and response to antimicrobial resistance is faced with scarce innovation. Efforts to develop new antibiotics are slow, and we need new tools, such as artificial intelligence, to accelerate the filling of technological gaps. Innovation and productive structures, notably in low- and middle-income countries, are underfunded, as is the effort to establish coordination of initiatives and networks. In addition, dependence on imports and possible discontinuity in the supply of antimicrobials due to health crises, trade sanctions, conflicts, or other reasons are risks that must be considered in the formulation and implementation of health policies.

Antimicrobial resistance deserves to become a priority not only in terms of plans, but also in terms of investments and concrete goals, and it is no longer a problem that greatly affects neglected populations and deepens social inequalities and perpetuates cycles of poverty. The 2024 Declaration is an important signal, recognizing multiple dimensions of the situation. This serious threat to human health, animal health, and the environment must be faced with collective action between different peoples and nations, courage, urgency, speed, offering equitable and sustainable solutions. There will be no equitable access without adequate funding and local innovation and production. Based on the Declaration, the level of political commitment must be as high as possible, aiming to mobilize skills and resources, such as monitoring and evaluation, as well as a thorough review of the dominant modes of production that create imbalances and inequities. Actions must strongly consider the needs of the low- and middle-income countries that most potently suffer from their impacts. Wars, migrations, and geoeconomic conflicts, such as the ones we are currently witnessing, add an extra layer to the problem of antimicrobial resistance ^12^ ^,^ ^13^. At this juncture, leadership and political will are key elements for countries and regions, especially the least developed, to have access to appropriate technologies, products, approaches, and tools, reacting to the historical injustices of unequal access.
