# Supplementary material for: Global commitment to combat antimicrobial resistance
Source: Cad Saude Publica. 2025 Apr 11;41(4):e00190924. doi: 10.1590/0102-311XEN190924 (PMC11996183; doi:10.1590/0102-311XEN190924)
Supplement: Translation

# Translation: Compromisso global para combater a resistência a antimicrobianos

**DOI:** 10.1590/0102-311XPT190924

**Authors:** Claudia Chamas

**Affiliations:** 1 Centro de Desenvolvimento Tecnológico em Saúde, Fundação Oswaldo Cruz, Rio de Janeiro, Brasil.; 2 Instituto Nacional de Ciência e Tecnologia de Inovação em Doenças de Populações Negligenciadas, Rio de Janeiro, Brasil.

## Introdução

A resistência a antimicrobianos ocorre quando micro-organismos (por exemplo, bactérias, vírus, fungos, parasitas) não respondem mais aos medicamentos formulados para eliminá-los, tornando esses tratamentos pouco eficazes para conter infecções e afetando procedimentos médicos que dependem dessas terapias para reduzir o risco de complicações ^1^. O problema exige abordagem mais potente ou custosa. Nos casos mais severos, as infecções podem até se tornar intratáveis, levando a mortes ou incapacidades de longa duração. Trata-se de ameaça à saúde global, pois o fenômeno não conhece fronteiras. Além disso, dificulta o cumprimento dos Objetivos de Desenvolvimento Sustentável, especialmente aqueles relacionados à saúde, à redução da pobreza, à segurança alimentar e ao meio ambiente. Fatores como falta de acesso à água potável, ao saneamento e à higiene, controle precário de infecções nas unidades de saúde e dificuldade de acesso a diagnósticos, medicamentos e vacinas agravam a situação, em particular em países de renda média e baixa.

Anualmente, cerca de 7,7 milhões de pessoas morrem devido a infecções bacterianas, sendo que 4,95 milhões dessas mortes estão relacionadas a patógenos resistentes a antibióticos. Boa parte da carga da resistência a antimicrobianos é enfrentada por países de renda média e baixa ^2^. Para abordar a situação, em 26 de setembro de 2024, em Nova York (Estados Unidos), a 79ª sessão da Assembleia Geral da Organização das Nações Unidas (ONU) realizou a segunda Reunião de Alto Nível sobre Resistência a Antimicrobianos [High-Level Meeting on Antimicrobial Resistance]. O encontro representou esforço renovado e sinalização importante dos países diante das crescentes ameaças representadas pela resistência a antimicrobianos ^2^. Na ocasião, líderes globais aprovaram a *Declaração Política da Reunião de Alto Nível sobre Resistência a Antimicrobianos* [Political Declaration of the High-level Meeting on Antimicrobial Resistance]. As negociações intergovernamentais que precederam essa declaração foram cofacilitadas por Malta e Barbados ^3^.

A declaração de 2024 não é pioneira, podemos citar iniciativas na esfera internacional que ajudaram a chegar ao atual nível de alerta e escopo: (i) em 2015, o *Plano de Ação Global sobre Resistência a Antimicrobianos* [Global Action Plan on Antimicrobial Resistance], adotado pela Assembleia Mundial da Saúde, esclareceu o cenário e as estratégias que deveriam ser abordadas por meio de ações locais e mundiais ^4^; (ii) em 2016, uma iniciativa de líderes na ONU - *Declaração Política da Reunião de Alto Nível sobre Resistência a Antimicrobianos* ^5^ ^,^ ^6^ - sinalizou a urgência para conter a disseminação de patógenos resistentes a antimicrobianos; (iii) em 2017, foi instalado o Grupo de Coordenação Interagencial sobre Resistência a Antimicrobianos [Interagency Coordination Group on Antimicrobial Resistance], convocado pelo Secretário-Geral da ONU, e que formulou recomendações para garantir ação global sustentada sobre a resistência a antimicrobianos, incluindo a melhoria da regulamentação, da resposta sustentada de Uma Só Saúde e dos investimentos em inovação ^7^; e (iv) em 2017, a declaração dos líderes do G20 - *Moldando um Mundo Interconectado* [Shaping an Interconnected World] - destacou a importância do fomento à pesquisa e ao desenvolvimento, em especial para patógenos prioritários identificados pela Organização Mundial da Saúde (OMS) e tuberculose ^8^.

Não obstante esse relevante conjunto de políticas, planos e declarações, a humanidade está longe de dispor de soluções adequadas. A resistência compromete a efetividade de antimicrobianos essenciais para a realização de cirurgias e o manejo de enfermidades. Ação global coordenada é um dos caminhos mais eficazes para aumentar a consciência e vigilância em torno do problema, que é um desafio à saúde, à segurança alimentar e ao desenvolvimento. A declaração de 2024 estabelece um compromisso significativo para alavancar ações e monitoramentos, além de mobilizar investimentos, ora insuficientes, sobretudo para países mais pobres.

O Banco Mundial estima que é necessário um investimento de USD 9 bilhões por ano para países de baixa e média renda. A instituição intergovernamental South Centre propõe um plano global para a mobilização de financiamento sustentável para a resistência a antimicrobianos, visando à implementação de planos de ação nacionais e orçamento para países em desenvolvimento ^9^ ^,^ ^10^.

## Pontos relevantes da declaração política da reunião de alto nível sobre resistência a antimicrobianos

A declaração envolve amplos compromissos nos principais aspectos do combate à resistência a antimicrobianos. Os líderes globais firmaram compromisso com a redução das mortes associadas ao problema em 10% até 2030, além de considerarem sua “natureza multifacetada e transversal” ^5^. Outros comprometimentos relevantes são:

(i) Governança: até 2030, todos os países devem desenvolver ou atualizar e implementar planos de ação nacionais multissetoriais sobre resistência a antimicrobianos; solicita-se que as organizações quadripartites (OMS, Organização das Nações Unidas para Alimentação e Agricultura, Organização Mundial da Saúde Animal e Programa das Nações Unidas para o Meio Ambiente) atualizem o *Plano de Ação Global sobre Resistência a Antimicrobianos* até 2026; a Secretaria Conjunta Quadripartite sobre Resistência a Antimicrobianos deve ser formalizada como o mecanismo central de coordenação; a cooperação da Secretaria com organizações multilaterais relevantes, como o Banco Mundial, o Fundo das Nações Unidas para a Infância e a Organização Mundial das Aduanas, é incentivada; e as organizações quadripartites são convidadas a estabelecer painel independente de evidências para ações contra a resistência a antimicrobianos em 2025.

(ii) Financiamento: pactuar com o financiamento sustentável para a implementação de planos de ação nacionais; comprometer-se com o financiamento sustentável para a implementação de planos de ação nacionais sobre resistência a antimicrobianos; fortalecer o financiamento por meio das estruturas existentes e mobilizar recursos por meio de canais nacionais, bilaterais e multilaterais, especialmente para países em desenvolvimento; e incentivar os mecanismos de financiamento existentes (por exemplo, Banco Mundial, Fundo Global de Luta contra Aids, Tuberculose e Malária, Aliança Mundial para Vacinas e Imunização etc.) para facilitar o acesso ao financiamento ou expandir seu escopo, incluindo investimentos em antimicrobianos eficazes, vacinas, pesquisa e desenvolvimento, diagnósticos.

(iii) Acesso: garantir o acesso equitativo a antimicrobianos, vacinas e diagnósticos nos países em desenvolvimento, em linha com as listas globais de medicamentos essenciais (lista de medicamentos essenciais da OMS, por exemplo).

(iv) Saúde humana: garantir que programas nacionais de prevenção e controle de infecções estejam em vigor por meio da implementação da Estratégia Global sobre Prevenção e Controle de Infecções da OMS, de 2023, da Agenda de Imunização 2030, da Estratégia de Água, Saneamento e Higiene da OMS 2018-2025, e do Plano de Ação Global de Segurança do Paciente da OMS 2021-2030, com o objetivo de atingir metas como 100% dos países com serviços básicos de água, saneamento, higiene e resíduos em todos os estabelecimentos de saúde e 90% dos países que atendem a todos os requisitos mínimos da OMS para programas de prevenção e controle de infecções em nível nacional até 2030; promover o fornecimento oportuno e equitativo de vacinas, diagnósticos e tratamentos de qualidade e acessíveis, inclusive antimicrobianos, e garantir seu uso adequado; e garantir, até 2030, que o uso de antibióticos do grupo de acesso da OMS seja ampliado em relação à meta global de 2023.

(v) Saúde animal e agricultura: esforços para reduzir, até 2030, a quantidade de antimicrobianos usados globalmente no sistema agroalimentar em relação ao nível atual, considerando os investimentos em saúde animal e vegetal para prevenir e controlar infecções, reduzindo o uso inadequado de antimicrobianos.

(vi) Pesquisa e desenvolvimento, inovação e fabricação: promover financiamento para a pesquisa e o desenvolvimento para lidar com a resistência a antimicrobianos, bem como parcerias entre os setores público e privado, reconhecendo a necessidade de aumentar a pesquisa e o desenvolvimento voltados para a saúde pública com base em equidade e acessibilidade; promover transferência de tecnologia e o *know-how* e incentivar a inovação e os compromissos com o licenciamento voluntário, sempre que possível, em acordos em que o financiamento público tenha sido investido na pesquisa de antimicrobianos, para fortalecer as capacidades locais e regionais de fabricação e o acesso eficaz a vacinas, terapias, diagnósticos e suprimentos essenciais; aumentar a transparência dos preços de medicamentos, vacinas, dispositivos médicos, diagnósticos, produtos de assistência, terapias baseadas em células e genes e outras tecnologias de saúde em toda a cadeia de valor; apoiar os países em desenvolvimento a fortalecer a produção local de vacinas, medicamentos, diagnósticos e outras tecnologias de saúde, para facilitar o acesso equitativo, reconhecendo que os altos preços impedem o combate à resistência a antimicrobianos.

## Inovação e produção: perspectivas para o futuro próximo

O último aspecto é de especial importância para países emergentes como o Brasil. A pandemia da COVID-19 nos mostrou como estruturas de inovação local e produção local são importantes não apenas para o adensamento da cadeia produtiva farmacêutica, mas para reduzir vulnerabilidades em contexto de restrições de insumos e produtos e rupturas nas cadeias globais de suprimentos. Em momentos de grave crise sanitária, sistemas robustos e resilientes salvam vidas. Retrocedendo um pouco no tempo, lembramos o que aconteceu durante a Guerra das Malvinas, em 1982. A Argentina sofreu corte de abastecimento de produtos estratégicos, como antibióticos. O Brasil pôde enviar remessas de produtos, pois contava com fabricação doméstica ^11^. Não faltam exemplos, mesmo sem guerras ou pandemias, para nos mostrar o quanto a capacidade de produção de antibióticos e outros produtos é incontornável para a segurança do país. O Brasil precisa estar preparado para o enfrentamento à resistência a antimicrobianos e outras situações adversas. Nesse contexto, não se pode menosprezar o investimento estatal, a coordenação global e a cooperação local e internacional, com vista a aumentar o leque de opções acessíveis de tratamentos, vacinas e diagnósticos.

Embora o conjunto de intervenções seja mais amplo, e ações de prevenção, protocolos adequados e uso racional de antibióticos, cuidado de saúde, vigilância e melhoria da infraestrutura de água e esgoto possam poupar inúmeras vidas e contribuir para a mitigação à resistência, detecção e resposta à resistência a antimicrobianos se defronta com inovação escassa. Os esforços para o desenvolvimento de novos antibióticos são lentos e precisamos de novas ferramentas, como inteligência artificial, para acelerar o preenchimento das lacunas tecnológicas. A inovação e as estruturas produtivas, notadamente em países de renda média e baixa, são subfinanciadas, assim como o afinco no estabelecimento de coordenação de iniciativas e redes. Ademais, a dependência de importações e eventual descontinuidade no abastecimento de antimicrobianos por causa de crises sanitárias, sanções comerciais, conflitos ou outro motivo são riscos que devem ser considerados na formulação e implementação de políticas de saúde.

A resistência a antimicrobianos merece se tornar prioridade não apenas em termos de planos, mas de investimentos e metas concretas, deixando de ser um problema que afeta sobremaneira populações negligenciadas e aprofunda desigualdades sociais e perpetua ciclos de pobreza. A declaração de 2024 é um importante sinalizador, reconhecendo múltiplas dimensões da situação. Essa grave ameaça à saúde humana, à saúde animal e ao meio ambiente deve ser enfrentada com ação coletiva entre os diferentes povos e nações, com coragem, urgência, velocidade, ofertando soluções equitativas e sustentáveis. Não haverá acesso equitativo sem financiamento adequado e inovação e produção local. A partir da declaração, o nível de compromisso político deve ser o mais elevado possível, com vista à mobilização de competências e recursos, bem como monitoramento e avaliação, além de profunda revisão nos modos de produção dominantes que geram desequilíbrios e inequidades. As ações devem considerar fortemente as necessidades dos países de renda média e baixa que sofrem seus impactos de maneira mais potente. Guerras, migrações e conflitos geoeconômicos, como os que presenciamos atualmente, adicionam uma camada extra ao problema da resistência a antimicrobianos ^12^ ^,^ ^13^. Nessa conjuntura, liderança e vontade política são elementos-chave para que países e regiões, especialmente os menos desenvolvidos, possam ter acesso a tecnologias, produtos, abordagens e ferramentas adequadas, reagindo às injustiças históricas do acesso desigual.
